# Human haematopoietic stem/progenitor cells express several functional sex hormone receptors

**DOI:** 10.1111/jcmm.12712

**Published:** 2015-10-30

**Authors:** Ahmed Abdelbaset‐Ismail, Malwina Suszynska, Sylwia Borkowska, Mateusz Adamiak, Janina Ratajczak, Magda Kucia, Mariusz Z. Ratajczak

**Affiliations:** ^1^ Stem Cell Institute at James Graham Brown Cancer CenterUniversity of LouisvilleLouisvilleKYUSA; ^2^ Department of Regenerative MedicineMedical University of WarsawWarszawaPoland; ^3^ Department of PhysiologyPomeranian Medical UniversitySzczecinPoland

**Keywords:** haematopoiesis, pituitary sex hormones, gonadal sex hormones, germline, haematopoietic development, VSELs

## Abstract

Evidence has accumulated that murine haematopoietic stem/progenitor cells (HSPCs) share several markers with the germline, a connection supported by recent reports that pituitary and gonadal sex hormones (SexHs) regulate development of murine HSPCs. It has also been reported that human HSPCs, like their murine counterparts, respond to certain SexHs (*e.g*. androgens). However, to better address the effects of SexHs, particularly pituitary SexHs, on human haematopoiesis, we tested for expression of receptors for pituitary SexHs, including follicle‐stimulating hormone (FSH), luteinizing hormone (LH), and prolactin (PRL), as well as the receptors for gonadal SexHs, including progesterone, oestrogens, and androgen, on HSPCs purified from human umbilical cord blood (UCB) and peripheral blood (PB). We then tested the functionality of these receptors in *ex vivo* signal transduction studies and *in vitro* clonogenic assays. In parallel, we tested the effect of SexHs on human mesenchymal stromal cells (MSCs). Finally, based on our observation that at least some of the UCB‐derived, CD45^−^ very small embryonic‐like stem cells (VSELs) become specified into CD45^+^ HSPCs, we also evaluated the expression of pituitary and gonadal SexH receptors on these cells. We report for the first time that human HSPCs and VSELs, like their murine counterparts, express pituitary and gonadal SexH receptors at the mRNA and protein levels. Most importantly, SexH if added to suboptimal doses of haematopoietic cytokines and growth factors enhance clonogenic growth of human HSPCs as well as directly stimulate proliferation of MSCs.

## Introduction

Development, migration, proliferation and differentiation of HSPCs is regulated in bone marrow (BM) by several well‐defined growth factors, cytokines, chemokines and bioactive lipids 1, 2, 3, 4. Evidence has accumulated that murine HSPCs share several markers with the germline, a connection supported recently by reports that pituitary and gonadal SexHs regulate the development of murine HSPCs 5, 6, 7. While the biological effects of SexHs on *in vivo* and *in vitro* murine haematopoiesis have recently been carefully evaluated by several groups, including our team 8, 9, 10, 11, 12, 13, the effects of these hormones, particularly pituitary SexHs, on human haematopoiesis requires further study.

For example, it is known that androgens can be successfully employed to treat aplastic anaemia in patients 14. On the other hand, it has been proposed that oestrogens and progesterone indirectly regulate human erythropoiesis by involving monocytes 15. By contrast, based on recent murine studies, it has been hypothesized that oestrogens play a role during pregnancy in which HSPCs respond to increased oxygen consumption and produce increasing numbers of erythrocytes 7. This latter hypothesis, however, still needs to be proven in humans. On the other hand, PRL compensates for erythropoietin (EPO) deficiency in patients on dialysis because of chronic kidney failure, and both *in vitro* and *in vivo* studies suggest that PRL can accelerate lymphoid and myeloid reconstitution and promote haematopoiesis 16, 17, 18. This multi‐lineage effect of human PRL makes it an attractive candidate in several clinical settings presenting with myelosuppression or immune deficiency 16. Moreover, oestrogens have been shown to regulate the final stages of megakaryopoiesis by facilitating proplatelet formation 19, 20, while progesterone promotes differentiation of T cells into T regulatory cells 12, 21.

In addition, the existence of developmentally early stem cells with broader specification in BM and UCB (generating a recent heated debate) has challenged the established hierarchy within the stem cell compartment 22, 23. As reported recently, murine HSPCs express functional pituitary FSH and LH receptors in addition to gonadal SexH receptors 8. Furthermore, following our observations that at least some murine BM‐derived, CD45^−^ VSELs become specified into CD45^+^ HSPCs 24, 25, we found that VSELs, like HSPCs, do express functional SexH receptors 8. Since at least some VSELs share several markers characteristic of migrating primordial germ cells (PGCs) 26, this observation sheds new light on the BM stem cell hierarchy and the potential link between murine VSELs, HSPCs and PGCs. Specifically, HSPCs might be specified at the time of embryogenesis from a population of migrating PGCs 22, 26, 27, later on from VSELs residing in foetal liver 28, 29, and in adults from VSELs in BM 24.

To shed more light on the role of SexHs in human haematopoiesis, we tested the expression of receptors for pituitary‐ and gonad‐derived SexHs on human UCB‐ and PB‐purified HSPCs and tested the functionality of these receptors in *ex vivo* signal transduction studies and clonogenic assays. In parallel, we tested the effect of SexHs on the proliferation of human MSCs. We also evaluated the expression of SexH receptors on human UCB‐derived CD133^+^ Lin^−^ CD45^−^ cell populations highly enriched in VSELs.

We report here for the first time that human CD45^+^ HSPCs and CD45^−^ VSELs, like their murine counterparts, express pituitary and gonadal SexH receptors at the mRNA and protein levels. Most importantly, SexH co‐stimulate clonogeneic growth of human HSPCs if added to suboptimal doses of haematopoietic cytokines and growth factors as well as directly stimulate proliferation of MSCs.

## Material and methods

### Isolation of human CD34^+^ population from peripheral blood

Low‐density mobilized and immobilized PB mononuclear cells (mPB‐MNCs and PB‐MNCs respectively) were harvested from consenting healthy donors. From these MNCs, cell populations enriched in CD34 markers were collected as described earlier 30.

### Isolation of CD34^+^ cells from umbilical cord blood

In some experiments, CD34^+^ cells from human UCB were also separated by immune‐mediated positive selection using anti‐CD34^+^ magnetic paramagnetic beads (Miltenyi Biotec GMBH, Bergisch Gladbach, Germany), according to the manufacturer's protocol. The purity of isolated CD34^+^ cells was >95%, as determined by fluorescence‐activated cell sorter (FACS; Beckman Coulter, Brea, CA, USA) analysis.

### Isolation of human UCB–derived HSPCs and VSELs

We used clinical‐grade UCB research units shipped from Cleveland Cord Blood Center for isolation of UCB‐HSPCs and UCB very small embryonic‐like stem cells (UCB‐VSELs). In brief, total nucleated cells (TNCs) were retrieved after lysing red blood cells twice, for 10 min. each time, at room temperature using hypotonic lysing buffer (BD PharmLyse, San Jose, CA, USA). Next, the TNCs were washed twice in RPMI‐1640 medium (GE Healthcare, South Logan, UT, USA) supplemented with 2% inactivated foetal bovine serum (FBS; Seradigm, Radnor, PA, USA). Using a cocktail of biotin‐conjugated monoclonal antibodies and anti‐biotin monoclonal antibodies conjugated to paramagnetic microbeads (Lineage Cell Depletion kit; Miltenyi Biotec GMBH), magnetic labelling of TNCs was performed, and the lineage‐negative (Lin^−^) cells were isolated by depletion of mature haematopoietic cells expressing a panel of lineage antigens using an autoMACS separator (Miltenyi Biotec GMBH). Afterwards, lineage^neg^ populations were stained with the following antibodies: anti‐CD45 (PE or V450, clone HI30) and anti‐CD34 (APC or PE, clone 581). After washing, the fluorochrome‐labelled cells were resuspended and sorted using a multiparameter live‐cell sorter (Beckton Dickinson, San Jose, CA, USA) to obtain populations enriched in HSPCs (Lin^−^/CD45^+^/CD34^+^) and VSELs (Lin^−^/CD45^−^/CD34^+^).

### Isolation and cultivation of human mesenchymal stromal cells

Mesenchymal stromal cells from UCB (hUCB–MSCs) were obtained from the CD34^−^ cell fraction after paramagnetic separation, as previously described. Directly after the separation process, the cells were centrifuged, washed with PBS, and then cultivated in DMEM (Corning Costar, Corning, NY, USA), supplemented with 20% FBS, 100 IU/ml penicillin, and 10 μg/ml streptomycin (both from Lonza, Walkersville, MD, USA) in a 5%‐CO_2_ incubator at 37°C. After 7 days of primary cultivation, fibroblast‐like cells were obtained. The medium was immediately exchanged and then every 3 days thereafter. At approximately 80% confluence, the cells were passaged using 0.25% trypsin (Corning Costar).

### Culture of primary human umbilical vein endothelial cells

Primary human umbilical vein endothelial cells (HUVECs) were purchased from American Type Culture Collection (ATCC, Manassas, VA, USA). HUVECs were cultured and grown in vascular cell basal medium (PCS‐100‐030; ATCC) supplemented with bovine brain extract (0.2%), recombinant human (rh) EGF (5 ng/ml), L‐glutamine (10 mM), heparin sulphate (0.75 IU/ml), hydrocortisone hemisuccinate (1 μg/ml), 2% FBS and ascorbic acid (50 μg/ml). All supplements were purchased from ATCC (#PCS‐100‐040). These cells were cultured at 37°C in a CO_2_ humidified atmosphere and detached from the growth plates using non‐enzymatic cell dissociation solution (Cellstriper; Corning Costar).

### Clonogenic assays *in vitro*

Isolated hUCB CD34^+^ cells obtained from healthy donors were subjected to colony assays in response to different SexHs. In brief, sorted CD34^+^ cells (4 × 10^5^ cells/ml) were resuspended in RPMI 1640 medium supplemented with 2% FBS (final concentration), and then mixed with methylcellulose base cultures (MethoCult HCC‐4230; StemCell Technologies, Inc., Vancouver, BC, Canada) supplemented with L‐glutamine (Lonza) and antibiotics. In clonogenic *in vitro* assays we employed 1/10 of optimal doses of haematopoietic cytokines and growth factors. Growth of colony‐forming unit‐granulocyte/macrophage (CFU‐GM) colonies was stimulated with rh interleukin‐3 (IL‐3, 1 ng/ml) and rh granulocyte/macrophage colony‐stimulating factor (GM‐CSF, 0.5 ng/ml). Growth of burst‐forming unit‐erythroid (BFU‐E) colonies was stimulated with rh EPO (0.2 IU/ml) and rh stem cell factor (SCF, 1 ng/ml). To examine CFU‐megakaryocytes (CFU‐meg), rh thrombopoietin (TPO, 5 ng/ml) and rh IL‐3 (1 ng/ml) were added to the base medium. To growth CFU‐Mix colonies cells 31 were stimulated by IL‐3 (1 ng/ml), G‐CSF (0.5 ng/ml), SCF (1 ng/ml), EpO (0.2 IU/ml) and TpO (5 ng/ml). All cytokines and growth factors were purchased from R&D Systems (Minneapolis, MN, USA). Cells in clonogenic assays were co‐stimulated with SexHs (FSH [5 IU/ml], LH [5 IU/ml], PRL [1 μg/ml], estradiol [0.1 μM], progesterone [0.1 μM] or androgen [danazol, 4 mg/ml]). The pituitary hormones were purchased from ProSpec (East Brunswick, NJ, USA), while the gonadal hormones were purchased from Sigma‐Aldrich (St. Louis, MO, USA). The cells maintained without any hormonal treatment were served as a control. Cultures were then incubated at 37°C in a fully humidified atmosphere supplemented with 5% CO_2_. Two weeks later, the colonies formed were scored under an inverted microscope.

### Preparation of conditioned media

We used UCB‐MSCs to obtain conditioned media (CM). UCB‐MSCs in passage–3 were seeded into 6‐well plates at a density of 10,000 cells per well. The cells were nourished with 20% FBS DMEM until they reached approximately 85% confluence. At this time, the cells were washed twice with PBS, and then 0.5% bovine serum albumin (BSA; Sigma‐Aldrich) in DMEM was added to the cells, either alone (control) or with FSH (10 IU/ml), LH (10 IU/ml) or PRL (2 μg/ml). At the same time, only media with SexHs were also incubated. After 24 hrs, all CM were separately harvested, centrifuged, filtered, and then stored at −80°C until use.

### Transwell migration assay

The Transwell migration assay was performed as follows. UCB–derived HSPCs were rendered quiescent by incubation in RPMI medium supplemented with 0.5% BSA for 5 hrs at 37°C and then seeded at a density of 10 × 10^4^ cells/100 μl per insert into the upper chambers of Transwell polycarbonate membrane inserts with 8‐μm pore size (Costar Transwell; Corning Costar). The lower modified Boyden's chambers contained different concentrations of FSH (2–20 IU/ml), LH (2–20 IU/ml), PRL (0.5–5 μg/ml), estradiol (0.1–1 μM) or progesterone (0.1–1 μM). Foetal bovine serum (10%) and BSA (0.5%) in RPMI 1640 medium were used as positive and negative controls respectively. After 3 hrs of stimulation at 37°C, the migrated cells were collected from the lower chambers and then scored using FACS analysis. For UCB‐MSC chemotaxis, cells were detached with 0.25% trypsin, starved for 24 hrs, and then seeded into the upper gelatin‐coated (0.5%) inserts at a density of 7 × 10^4^ cells/insert in 100 μl. Pre‐warmed culture medium containing SexHs (FSH [10 IU/ml], LH [10 IU/ml], PRL [2 μg/ml] or estradiol [0.1 μM]) was added to the lower chambers. After 48 hrs, the inserts were collected from the Transwell supports. The cells that had migrated to the lower side of the membrane were fixed and stained with Hema–3 protocol (Fisher Scientific, Pittsburgh, PA, USA) according to the manufacturer's instructions and then counted using an inverted microscope.

### Adhesion of haematopoietic cells and MSCs to fibronectin

Umbilical cord blood‐derived HSPCs and MSCs were made quiescent for 5 and 8 hrs, respectively, with 0.5% BSA in a humidified atmosphere of 5% CO_2_ at 37°C. Next, cells were stimulated with FSH (10 IU/ml), LH (10 IU/ml), PRL (2 μg/ml), estradiol (0.1 μM) or 0.5% BSA in RPMI 1640 medium for 5 min. at 37°C. Cells were added directly onto the protein‐coated wells (3 × 10^3^ cells/well) in 96‐well plates for 5 min. The wells were coated with fibronectin (Sigma‐Aldrich; 10 μg/ml) overnight at 4°C and blocked with BSA for 2 hrs before starting the experiment. Following stimulation, the plates were vigorously washed 3 times, and adherent cells were counted under an inverted microscope.

### Signal transduction studies

Western blots were performed on extracts prepared from quiescent CD34^+^ HSPCs and MSCs (2 × 10^6^ cells). The cells were stimulated with either SexHs or BSA, as indicated, for 5 min. at 37°C and then lysed (for 20 min.) on ice in RIPA lysis buffer containing protease and phosphatase inhibitors (Santa Cruz Biotech, Santa Cruz, CA, USA). Next, the extracted proteins were separated on a 4–12% SDS‐PAGE gel, and the fractionated proteins were then transferred to a Polyvinylidene difluoride (PVDF) membrane. Phosphorylation of the intracellular kinases p44⁄42 mitogen‐activated protein kinase (phospho‐p44/42 MAPK) and AKT were detected using anti‐phospho‐p44/42 MAPK (Thr202/Tyr204; clone no. 9101) and anti‐phospho‐AKT (Ser473; clone no. 9271) rabbit polyclonal antibodies (Cell Signaling, Danvers, MA, USA) followed by horseradish peroxidase‐conjugated goat anti‐rabbit IgG as a secondary antibody (Santa Cruz Biotech). To ensure equal protein loading in all lanes, blots were subjected to stripping and reprobing with appropriate anti‐p42/44 MAPK (clone no. 9102; Cell Signaling) and anti‐AKT monoclonal antibodies (clone no. 9272; Cell Signaling). All membranes were treated with an enhanced chemiluminescence reagent (Amersham Life Sciences, Amersham, UK), dried, and subsequently exposed to film (Hyperfilm; Amersham Life Sciences). Quantification of the densities of obtained blots was performed with ImageJ software (National Institutes of Health, Bethesda, MD, USA).

### RNA isolation and RT‐PCR

Total RNA was extracted and purified from hUCB‐derived CD34^+^ Lin^−^ CD45^+^ HSPCs and MSCs using the RNeasy Mini kit (Qiagen Inc., Valencia, CA, USA) after treatment with DNase I (QIAGEN Inc., Valencia, CA, USA). In the case of VSELs, the total RNA was extracted using Trizol, as described previously 32. The mRNA (200 ng) was next reverse‐transcribed into cDNA using Taqman Reverse Transcription reagents (Applied Biosystem, Branchburg, NJ, USA), according to the manufacturer's instructions. Amplification of synthesized cDNA fragments was carried out using Amplitaq Gold polymerase (Applied Biosystems) for 1 cycle of 8 min. at 95°C, 2 cycles of 2 min. at 95°C, 1 min. at 60°C, 1 min. at 72°C and then for 40 cycles of 30 sec. at 95°C, 1 min. at 60°C, 1 min. at 72°C, and 1 cycle of 10 min. at 72°C. The human sequence‐specific primers are shown in Figure S1. All primers were designed using the NCBI/Primer‐Blast program, as at least one primer included an exon–intron boundary. At the end of the PCR reaction, the PCR products were analysed by 2% agarose gel electrophoresis.

### Real‐time quantitative PCR

To evaluate the role of FSH in the regulation of genes expressed actively in angiogenesis, MSCs were kept in culture, either unstimulated or stimulated with FSH, for 12–24 hrs. At various time‐points, the cells were collected, and total RNA was extracted and purified using the RNeasy Mini kit (Qiagen Inc.) after treatment with DNase I (Qiagen Inc.). The mRNA was then reverse‐transcribed into cDNA using Taqman Reverse Transcription Reagents (Applied Biosystems), according to the manufacturer's instructions. Quantitative assessment of mRNA levels of target genes was performed by RQ‐PCR using an ABI Prism Fast 7500 sequence detection system (Applied Biosystems). The cDNA templates were amplified using SYBR Green PCR master mix (Applied Biosystems), and specific primers (hVEGF2 R: forward, 5′‐ggtctcgattggatggcagtag‐3′, reverse, 5′‐cacccatggcagaaggagga‐3′; β2 microglobulin R: forward, 5′‐aatgcggcatcttcaaacct‐3′, reverse, 5′‐tgactttgtcacagcccaagata‐3′). These primers sequences were designed with Primer Express software (Applied Biosystems). The threshold cycle (Ct), the cycle number at which the fluorescence of the amplified gene reached a fixed threshold, was subsequently determined, and the relative quantification of the expression level of target genes was performed with the 2^−ΔΔCt^ method.

### Immunostaining of the isolated cells

Human UCB‐derived Lin^−^/CD133^+^/CD45^−^ cells (VSELs), and corresponding Lin^−^/CD133^+^/CD45^+^ cells (enriched for HSPCs), and MSCs were plated, fixed in 3.7% paraformaldehyde for 15 min. at 4°C, and then permeabilized with 0.1% Triton X‐100 for 5 min. After blocking with 2.5% BSA, the cells were subjected to immunostaining with the following primary antibodies: follicle‐stimulating hormone receptor (FSHR, 1:200, rabbit polyclonal antibody; Santa Cruz Biotech), luteinizing hormone/choriogonadotropin receptor (LHR, 1:200, rabbit polyclonal antibody; Santa Cruz Biotech), androgen receptor (1:50, rabbit polyclonal antibody; Neomarkers; Fremont, CA, USA), and oestrogen receptor alpha (1:500, mouse monoclonal IgG antibody; ThermoScientific; Grand Island, NY, USA). These antibodies were diluted in 2.5% BSA and incubated with the cells for 1 hr 15 min. at 37°C. Appropriate Alexa Fluor 594 Goat Anti‐Rabbit IgG and Alexa Fluor 488 Goat Anti‐Mouse IgG were used as secondary antibodies (1:400, all from Invitrogen, Life technology, Carlsbad, CA, USA), and incubated with the cells for staining for 75 min. at 37°C. In control experiments, cells were stained with secondary antibodies only. In all experiments, the nuclei were labelled with DAPI, and the fluorescence images were collected with a FV100 confocal laser‐scanning microscope (Olympus America Inc., Center Valley, PA, USA).

### *In vitro* CFU‐fibroblasts assay

In order to evaluate the effect of SexHs on fibroblast colony formation, hUCB‐MSCs were collected after trypsinization and then seeded onto 6‐well plates at a low cell density (30,000 cells per well) in 20% FBS DMEM and incubated at 37°C in a humidified atmosphere of 5% CO_2_. After 2 hrs, the cells were stimulated with SexHs at the dosages indicated. Fresh medium containing SexHs was added to the cells every 24 hrs for 10 days, after which the medium was removed and the cells subjected to Hema–3 staining protocol, and the fibroblast colony formation in all cell groups was then evaluated and counted.

### *In vitro* angiogenesis assay

The tube‐formation assay was performed on a synthetic Matrigel basement membrane with reduced growth factors (Matrigel; BD Bioscience, San Jose, CA, USA). In brief, the Matrigel was thawed overnight at 4°C. Twenty‐four‐well plates were coated with matrix and then polymerized for 1 hr at 37°C. Afterwards, HUVECs (60,000 cells/well) were resuspended in either serum‐reduced medium containing 0.5% BSA in DMEM medium (negative control), medium containing FGF2 (50 ng/ml; positive control), CM from unstimulated hUCB‐MSCs, medium supplemented with FSH (10 IU/ml), or CM collected from stimulated MSCs with the same concentration of FSH. The cells were seeded on the polymerized Matrigel matrix in duplicate and kept in a humidified environment of 5% CO_2_ at 37°C. After 2–4 hrs, the cultures were evaluated for capillary‐like tube formation, the identical fields in each well were photographed, and the formed tubes in each group were then scored.

### Statistical analysis

All data obtained are presented as mean ± S.D. Statistical analysis of the data was done by one‐way anova with post hoc Tukey's test using GraphPad Prism 5.0 program (GraphPad Software, Inc., La Jolla, CA, USA) with *P* < 0.05 and *P* < 0.01 considered significant.

## Results

### Human HSPCs express functional gonadal and pituitary sex hormone receptors

**Figure 1:**
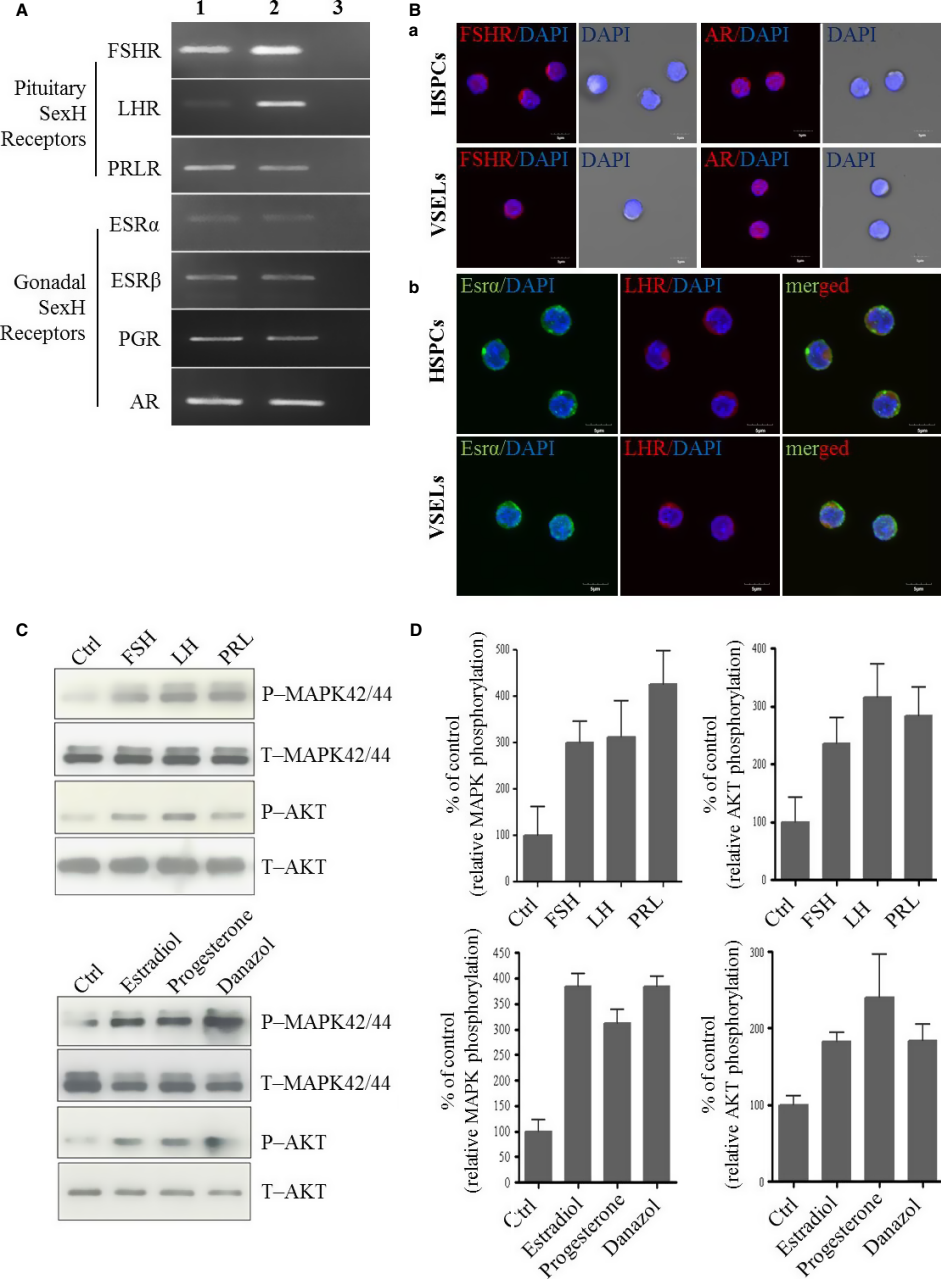
Human HSPCs and VSELs purified from umbilical blood (UCB) express functional SexH receptors. (**A**) RT‐PCR (n = 2) results for SexH (pituitary and gonadal) receptor expression in purified mRNA samples from sorted HSPCs (CD34^+^ Lin^−^ CD45^+^, Lane 1) and VSELs (CD34^+^ Lin^−^ CD45^−^, Lane 2) isolated from human UCB. Samples with water only instead of cDNA were used as negative controls (Lane 3). A representative agarose gel of the RT‐PCR amplicons is shown. (**B**) Expression of the pituitary SexH receptors FSHR (a) and LHR (b) and the gonadal SexH receptors AR (a) and Esrα (b) was detected on both human CD133^+^ VSELs and corresponding CD133^+^ enriched for HSPCs by immunofluorescence staining (n = 2), as described in Materials and Methods. (**C**) The effect of pituitary and gonadal SexHs on phosphorylation of p42/44 MAPK and AKT (Ser473) in CD34^+^ HSPCs. These cells were enriched from UCB by immunomagnetic microbeads and starved for 5 hrs in RPMI medium containing 0.5% BSA in an incubator and afterwards stimulated by SexHs for 5 min. before collecting protein lysates. One set of representative blots out of two is shown. (**D**) Densitometric analysis of blots shown in (C). The experiment was repeated twice on isolated cells with similar results, and representative images are shown. An example of control staining is shown in Figure S3. Abbreviations: FSHR: follicle‐stimulating hormone receptor; LHR: luteinizing hormone/choriogonadotropin receptor; AR: androgen receptor; ESR: oestrogen receptor; PGR: progesterone receptor; SexHs: sex hormones; MNCs: mononuclear cells; VSELs: very small embryonic‐like stem cells; HSPCs: haematopoietic stem/progenitor cells.

It has been reported that murine HSPCs express several SexH receptors 8, and we became interested in whether SexH receptors are expressed by human HSPCs as well. Figure 1A shows that two FACS‐sorted populations, human CD34^+^ Lin^−^ CD45^+^ cells, which are highly enriched for HSCs, and small CD34^+^ Lin^−^ CD45^−^ cells, which are highly enriched for VSELs, express mRNA for all pituitary and gonadal SexH receptors. We obtained similar mRNA expression for human CD34^+^ HSPCs isolated from normal and mobilized PB (Fig. S2). Expression of SexH receptors at the protein level on human HSPCs was subsequently confirmed in human UCB‐purified CD133^+^ cells by immunostaining (Fig. 1B).

Next, we became interested in whether human CD34^+^ cells sorted from UCB respond to stimulation by SexHs by phosphorylation of MAPKp42/44 and AKT. Figure 1C shows that human CD34^+^ cells respond to stimulation by both pituitary and gonadal SexHs, and these results are quantified in Figure 1D.

### SexHs stimulate *in vitro* growth of human clonogenic progenitors

**Figure 2:**
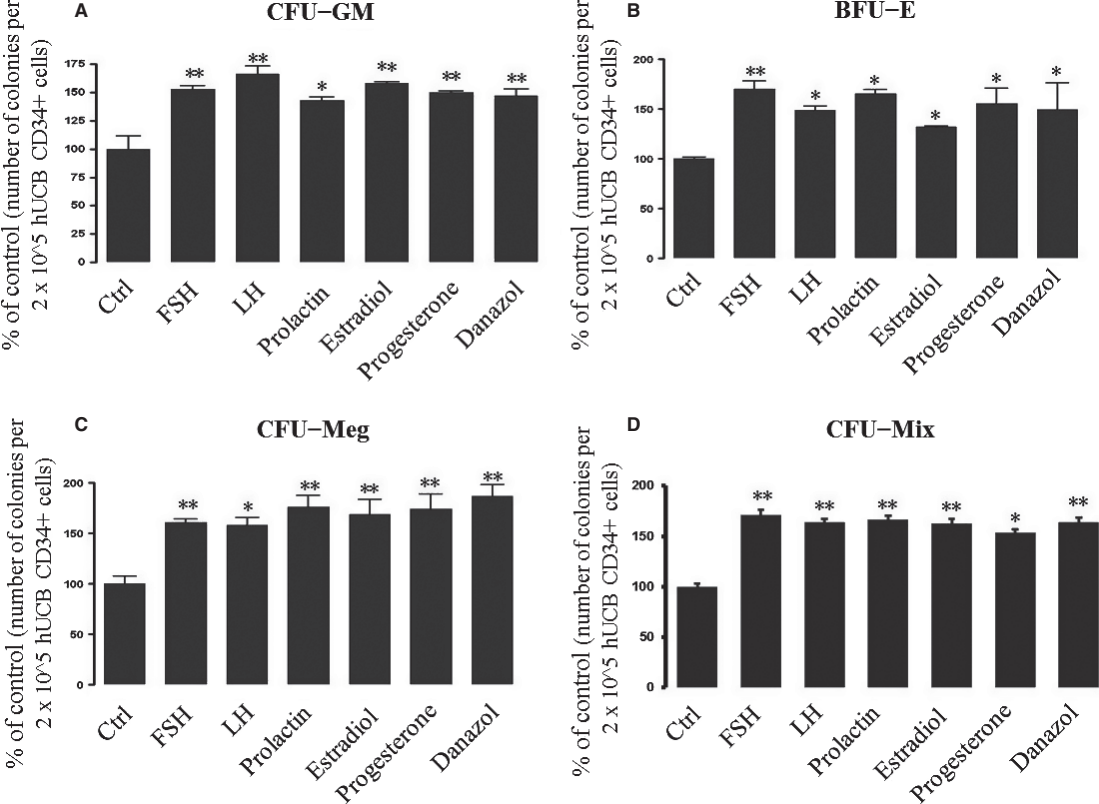
Increase in the number of clonogenic CFU‐GM (**A**), BFU‐E (**B**), CFU‐Meg (**C**), and CFU‐Mix (**D**) progenitors *in vitro* after co‐stimulation of human UCB CD34^+^ progenitors with SexHs. SexHs co‐stimulate *in vitro* proliferation of human UCB CD34^+^ cells in methylcellulose‐based cultures containing suboptimal doses (1/10 of optimal doses) of growth factors/cytokines and the indicated doses of pituitary and gonadal SexHs. The number of colonies formed in the absence of SexHs was considered to be 100%. Data are combined from four independent experiments and means ± S.D. are shown. *P < 0.05 and **P < 0.01 are considered significant compared with the control group. Abbreviations: BFU‐E: erythrocyte burst‐forming units; CFU‐GM: granulocyte/macrophage colony‐forming units; CFU‐Meg: megakaryocytic colony‐forming units.

Next, we asked whether human clonogenic progenitors respond to stimulation by SexHs as previously observed for murine cells 8. To address this question, we performed *in vitro* assays in which sorted CD34^+^ cells isolated from UCB were stimulated with suboptimal doses (1/10 of the optimal dose) of growth factors and cytokines in the presence or absence of pituitary or gonadal SexHs (Fig. 2) and found that all SexHs increased clonogenic growth of human BFU‐E, CFU‐GM, CFU‐Meg and more primitive CFU‐Mix progenitors (*P* < 0.05).

At the same time, while CD34^+^ cells responded to SexH stimulation by phosphorylation of MAPKp42/44 and AKT (Fig. 1C and D), to our surprise, we did not observe any effect of SexHs on migration or adhesion of clonogenic CD34^+^ cells (data not shown).

### Human mesenchymal stromal cells express several functional SexH receptors

**Figure 3:**
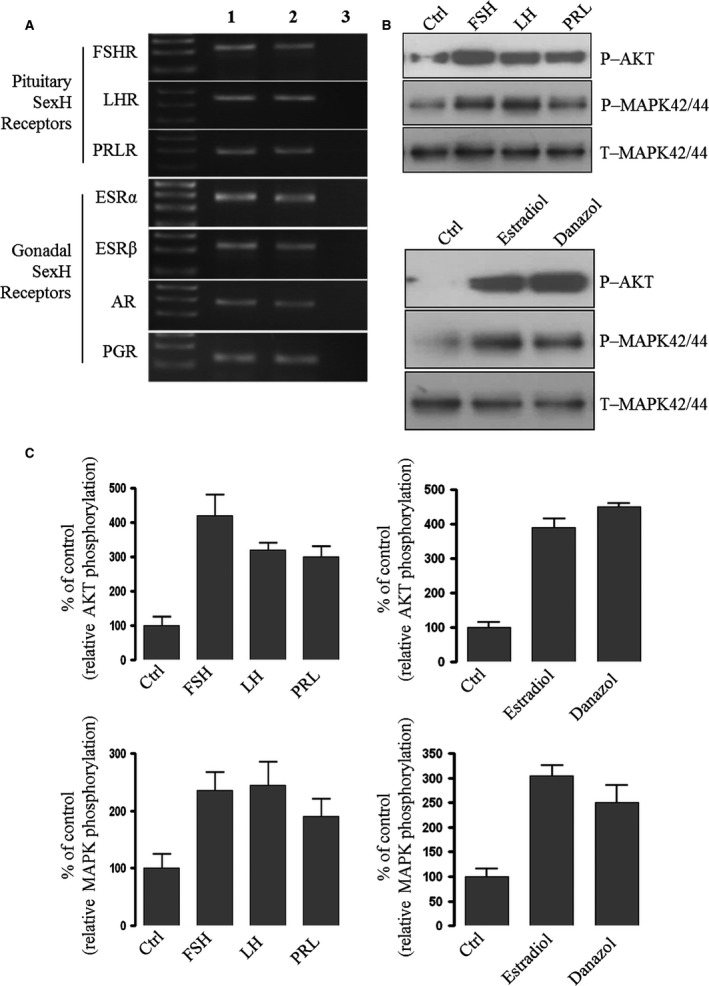
Human mesenchymal stromal cells (hMSCs) purified from umbilical cord blood (UCB) express functional SexH receptors. (**A**) RT‐PCR (n = 2) for SexH (pituitary and gonadal) receptor expression in purified mRNA samples from cultured UCB‐derived MSCs (Lane 1) and human ovarian cancer cell line cells (hOCCs) employed as positive control (Lane 2). Samples with water only instead of cDNA template were used as negative controls (Lane 3). A representative agarose gel of the RT‐PCR amplicons is shown. Expression of these SexH receptors was also confirmed on human MSCs by immunofluorescence staining (Fig. S3) (n = 2). (**B**) The effect of pituitary and gonadal SexHs on the phosphorylation of p42/44 MAPK and AKT (Ser473) in human UCB‐derived MSCs, which were starved for 16 hrs in DMEM medium containing 0.5% BSA in an incubator and afterwards stimulated by SexHs (FSH [10 IU/ml], LH [10 IU/ml], PRL [2 μg/ml], estradiol [0.1 μM] or danazol [4 mg/ml]) for 5 min. before collecting protein lysates. One set of representative blots out of two is shown. (**C**) Densitometric analysis of blots shown in (**B**). The experiment was repeated twice on isolated cells with similar results, and representative images are shown.

Since MSCs play an important role in haematopoiesis, we became interested in the role of SexHs in these cells. First, we evaluated the expression of SexH receptors on human UCB‐derived MSCs at the mRNA level. Figure 3A shows that all pituitary and gonadal SexH receptors evaluated in our studies were expressed by human MSCs, which was subsequently confirmed at the protein level (Fig. S3). Moreover, our signal transduction experiments revealed that these receptors are functional, as UCB‐derived MSCs respond to SexH stimulation by MAPKp42/44 and AKT phosphorylation (Fig. 3B and C).

**Figure 4:**
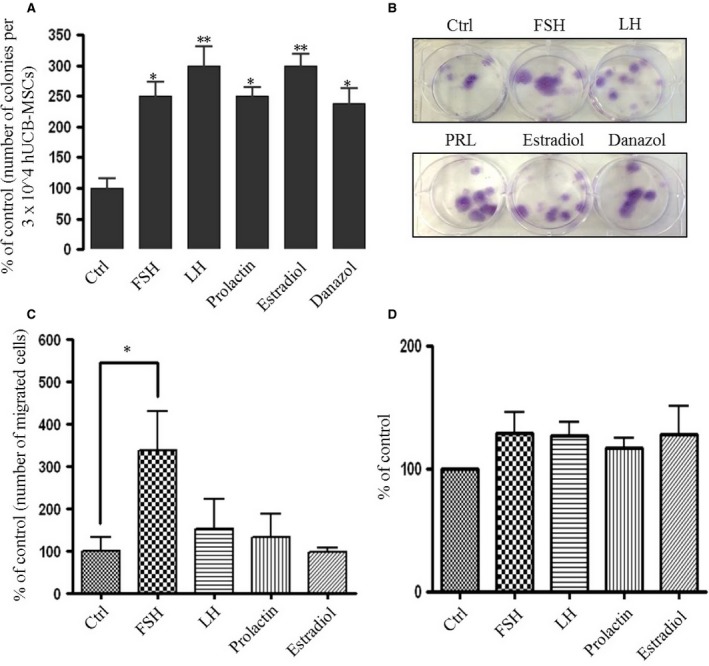
Human MSCs proliferate *in vitro* after stimulation with SexHs. (**A**) Changes in the number of fibroblasts colony‐forming units (CFU‐F) *in vitro* from human UCB‐derived MSCs plated at limiting dilution conditions in the presence of the indicated SexHs for 10 days. MSCs were seeded into six‐well culture plates in DMEM medium supplied with 20% FBS, alone or with SexHs (FSH [10 IU/ml], LH [10 IU/ml], PRL [2 μg/ml], estradiol [0.1 μM] or danazol [4 mg/ml]). After 10 days, the cells were washed with PBS twice, stained using a Hema–3 staining kit and the colonies were counted. Experiments were repeated thrice with similar results. The data are normalized to CFU–F of non‐stimulated cells, which was assumed to be 100%. Data from two separate experiments (each in duplicate) are presented as means ± S.D., and *P < 0.05 and **P < 0.05 are significant. (**B**) Representative images of stained CFU‐F colonies are shown. (**C**) The effect of pituitary and gonadal hormones on the migration of human MSCs. The data are normalized to chemotaxis in response to medium alone, which was assumed to be 100%. Data from two separate experiments (each in triplicate) are combined together and shown as means ± S.D. *P < 0.05 *versus* the control. (**D**) The effect of pituitary and gonadal hormones on the adhesion of human MSCs to fibronectin. The data are normalized to adhesion of non‐stimulated cells, which was assumed to be 100%. Data from two separate experiments (each in triplicate) are combined together and shown as means ± S.D.

Since SexHs activated human MSC phosphorylation of MAPKp42/44 and AKT, we investigated whether they stimulate proliferation of these cells. As demonstrated in Figure 4A and B, SexHs stimulated proliferation of MSC‐derived CFU‐F colonies.

Next, we employed Transwell chemotactic cell migration assays and found that, of all the SexHs tested, only FSH strongly chemo‐attracted UCB‐derived MSCs (Fig. 4C). Furthermore, FSH, like other SexHs, slightly enhanced adhesion of MSCs (Fig. 4D).

**Figure 5:**
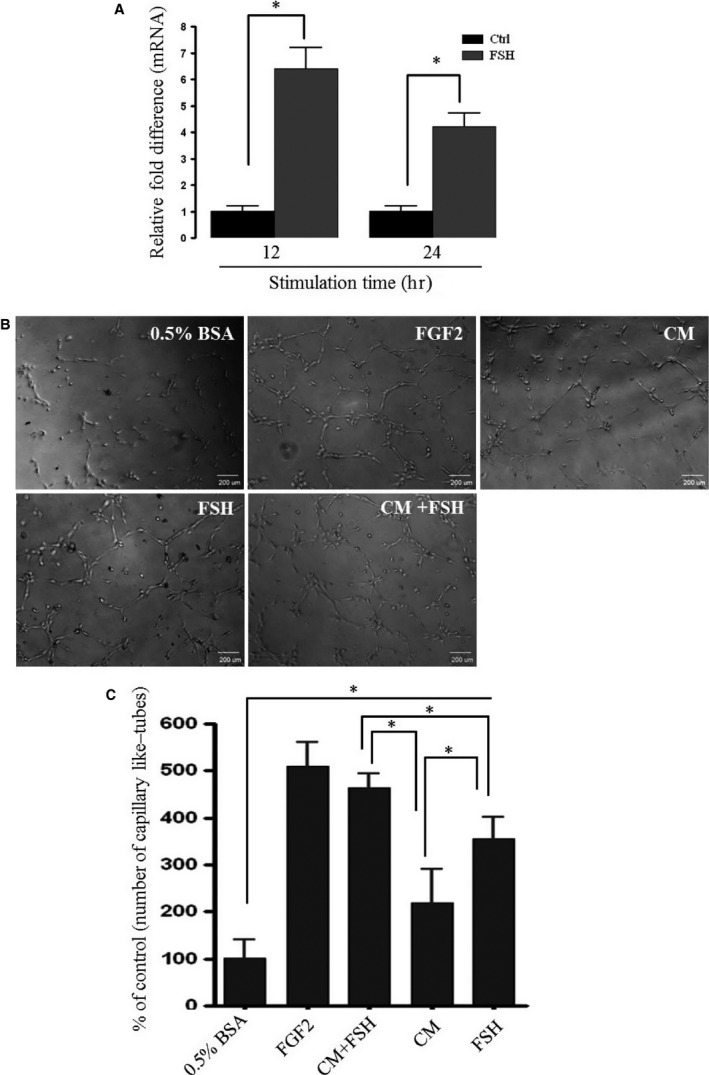
MSCs stimulated by FSH promote angiogenesis. (**A**) An increase in expression of hVEGF2 at mRNA level in human MSCs stimulated by FSH (10 IU/ml) for 12 and 24 hrs *in vitro* compared with unstimulated control cells. (**B**) Capillary‐like structure formation in 3D‐Matrigel cultures *in vitro* (tube‐formation assay). The assay was done on HUVECs seeded on Matrigel cultures in the presence of either conditioned medium (CM) from unstimulated MSCs, FSH (10 IU/ml) alone or CM from MSCs stimulated with the same concentration of FSH. As a positive control, human recombinant FGF2 (100 ng/ml) was used. (**C**) The results are shown relative to tube formation by HUVECs stimulated with 0.5% BSA only, which was assumed to be 100%. The experiment was repeated twice independently with the same results, and the combined data are shown. For statistical comparisons, a one‐way anova was carried out and means ± S.D. are shown. Significance level: *P < 0.05 *versus* control.

Finally, we evaluated the effect of SexHs on expression of selected growth factors and chemokines involved in angiogenesis in UCB‐derived MSCs. Our RQ‐PCR results reveal that FSH strongly up‐regulates expression of VEGF (Fig. 5A). Therefore, to address the potential effect of FSH‐stimulated MSCs on endothelium, we performed a tube‐formation assay employing HUVECs. Figure 5B and C demonstrates that tube formation by HUVECs was significantly enhanced in the presence of CM isolated from cells stimulated by FSH as well as by FSH alone.

## Discussion

The salient observation of our work is that functional pituitary and gonadal SexHs are expressed by human HSPCs and that SexHs directly enhance clonogenic growth of human CFU‐GM, BFU‐E and CFU‐Meg progenitors. Moreover, we demonstrated for the first time that human VSELs, like their murine counterparts 8, also express several SexH receptors at both the mRNA and protein levels.

These results are relevant for a few reasons. First, they suggest a developmental connection between the germline, particularly migrating PGCs, and haematopoiesis 22, 23, 26, 27, 33, 34, 35. In support of this intriguing possibility, specification of the first primitive HSPCs in the yolk sac blood islands as well as the origin of definitive HSPCs in the aorta–gonado–mesonephros region are chronologically and anatomically correlated with the developmental migration of PGCs in extra‐ and intra‐embryonic tissues 4, 22, 23, 26, 33. Based on these observations, it has been postulated that a subpopulation of cells derived from PGCs could contribute during the earliest stages of embryogenesis to the population of hemangioblasts in the yolk sac and, later on before entering the genital ridges, could contribute to the population of HSPCs in the hemangiopoietic endothelium of aorta 36, 37, 38. Of course, this intriguing concept, which is supported by some developmental studies, needs further experimental support. Nevertheless, it has been demonstrated that murine PGCs are able to give rise to HSPCs *in vitro* 27, 34 and *in vivo* 38. Similarly, haematopoietic development was observed in murine 34, 35, 39 and human 40, 41 teratocarcinoma cell lines. Furthermore, several papers have described the sharing of chromosomal aberrations between germline tumours and leukaemias or lymphomas, which suggests their shared clonal origin 40, 41, 42, 43. Moreover, as we recently reported, murine and human germline cells share with HSPCs a functional erythropoietin receptor 44. Accordingly, human and murine germline‐derived teratocarcinoma cell lines as well as ovarian cancer cell lines respond to EPO by chemotaxis, increased adhesion and phosphorylation of MAPKp42/44 and AKT 44. Finally, the transcription factor Sall4 has been reported to play an important role in both haematopoiesis and germline development 45.

Our data also raise another intriguing question. Since it has been demonstrated that murine 24 and human 25, 46 VSELs become specified into HSPCs in appropriate co‐culture conditions, the question is whether VSELs are precursors for the haematopoietic lineage 24, 25, 47, 48, as it has been already demonstrated by others that they are precursors of MSCs in BM 49. The presence of primitive haematopoietic precursors in adult BM that do not meet the phenotypic criteria of ‘classical’ long‐term repopulating haematopoietic cells (LT‐HSCs) has been proposed by several authors, and such cells have been given different operational names 22. Some of them have been reported to be CD45^−^, as have our VSELs 8. Taking into consideration that VSELs also express several genes characteristic of endothelial cells (*e.g*. Flk‐1), further work is needed to explore the possible relationship between VSELs and hemangioblasts, which populate the foetal liver during embryogenesis and adult BM later 4, 8, 29. Interestingly, as recently reported human VSELs isolated from PB from patients with critical limb ischaemia are endowed with remarkable *in vivo* angiopoietic potential 50. It is also tempting to conjecture that VSELs are the missing connection between PGCs and LT‐HSCs, and, in fact, it has been reported that VSELs express several genes and markers characteristic of migrating PGCs, including murine vasa homologue 26.

Finally, our data show a functional link between haematopoiesis, MSCs and angiogenesis in the responsiveness of BM cells to SexHs. First, we have demonstrated for the first time the effect of pituitary SexHs, such as FSH and LH on human haematopoiesis. These observations complete an old study showing that hypophysiotropic hormones, such as FSH‐releasing protein [known as activin and FSH‐release inhibiting protein (inhibin)], regulate the *in vitro* development of erythroid colonies 51. In addition we propose here that FSH and LH, like other SexHs 14, could be employed to treat aplastic anaemias or to accelerate haematopoietic recovery, for example, in irradiated victims. Moreover, as FSH is up‐regulated in older patients as a result of age‐dependent gonadal dysfunction 52, 53, its possible role in co‐facilitating development of myeloid leukaemia is an interesting question to explore. Therefore, further studies are needed to elucidate the effects of SexHs on malignant haematopoiesis, and our preliminary data demonstrate that SexHs modulate several processes in human leukaemic cells (unpublished data; Novel evidence that pituitary gonadotropins directly stimulate human leukemic cells‐studies of myeloid cell lines and primary patient AML and CML cells, A. Abdelbaset‐Ismail, S. Borkowska, A. Janowska‐Wieczorek, T. Tonn, C. Rodriguez, M. Moniuszko, L. Bolkun, J. Kloczko, J. Ratajczak, M.Z. Ratajczak, M. Kucia, under revision).

We also demonstrated in this paper that normal human MSCs express several functional SexH receptors and respond by proliferation to SexH simulation. The observed pro‐angiopoietic effect of CM from FSH‐stimulated MSCs and up‐regulation of VEGF in these cells sheds additional light on SexH‐mediated interactions between MSCs and endothelium. FSH has already been reported to stimulate murine endothelial cells 54, and our results support the possibility that such an effect occurs also in human cells. Moreover, our recent Gene Array data on MSCs stimulated by FSH revealed up‐regulation of several genes involved in angiogenesis and pro‐angiopoietic factors signalling (unpublished data). This all together demonstrates pleiotropic effects of SexH on BM microenvironment and expands our knowledge on novel factors that modulate haematopoiesis, MSCs biology and angiogenesis.

In conclusion, we report for the first time that both human HSPCs and VSELs, like their murine counterparts, express the entire panel of pituitary and gonadal SexH receptors. Most importantly, however, SexHs alone do not stimulate *in vitro* proliferation of HSPCs, they co‐stimulate clonogenic growth of human HSPCs if added to suboptimal doses of haematopoietic cytokines and growth factors. SexHs also stimulate proliferation of MSCs and increase their pro‐angiopoietic potential. Finally, our results also shed more light on the possible developmental link between PGCs, VSELs, and HSPCs—an intriguing hypothesis that needs further study, and results obtained with normal HSPCs lend support to studying the effects of SexHs in malignant haematopoiesis.

## Conflicts of interest

The authors declare that there are no conflicts of interest.

## Supporting information


**Figure S1** Sequences of primers employed to detect human SexH receptors at the mRNA level by RT‐PCR.

**Figure S2** Human CD34^+^ cells purified from peripheral blood (PB) express functional SexH receptors. RT‐PCR (n = 2) for the expression of pituitary and gonadal SexH receptors after mRNA purification from PB CD34^+^ cells from a normal donor (Lane 1) and a mobilized donor (Lane 2). Samples containing water only instead of cDNA were used as negative controls (Lane 3). Representative agarose gel of RT‐PCR amplicons is shown.

**Figure S3** Expression of two pituitary SexH receptors, FSHR and LHR, and two gonadal SexH receptors, AR and ESR, was detected on human MSCs by immunofluorescence staining (**A**) (n = 2). An example of control staining showing that a secondary antibody conjugated with Alexa 488 or Alexa 594 did not bind to cells if the primary antibody was not employed (**B**).
