# Supplementary material for: Human haematopoietic stem/progenitor cells express several functional sex hormone receptors
Source: J Cell Mol Med. 2015 Oct 30;20(1):134–46. doi: 10.1111/jcmm.12712 (PMC4717849; doi:10.1111/jcmm.12712)
Supplement: Supplementary file 1 — Figure S1 Sequences of primers employed to detect human SexH receptors at the mRNA level by RT‐PCR. [file JCMM-20-134-s001.pdf]

HUMAN SEX HORMONE RECEPTOR	PRIMER SEQUENCES		PRODUCT LENGTH (bp)
FSH–R	sense	5′-gcttctgagatctgtggaggtt-3′	231
	antisense	5′-ggacaaacctcagttcaatggc-3′	
LH–R	sense	5′-cagaggccgtccaagacac-3′	330
	antisense	5′-atgctccgggctcaatgtat-3′	
Prolactin–R	sense	5′-gagcttcttctcacagagcca-3′	291
	antisense	5′-aagttcacttcagggttcatgtgg-3′	
Androgen–R	sense	5′-cgacttcaccgcacctgatg-3′	296
	antisense	5′-acttctgtttcccttcagcgg-3′	
Estrogen–Rα	sense	5′-aggtgccctactacctggag-3′	397
	antisense	5′-cggtcttttcgtatcccacct-3′	
Estrogen–Rβ	sense	5′-aatggtgaagtgtggctccc-3′	345
	antisense	5′-acttggtcgaacaggctgag-3′	
Progesterone–R	sense	5′-tcaactacctgaggccggat-3′	336
	antisense	5′-cagcatccagtgctctcaca-3′	
