# Supplementary figures and images for: Human haematopoietic stem/progenitor cells express several functional sex hormone receptors
Source: J Cell Mol Med. 2015 Oct 30;20(1):134–46. doi: 10.1111/jcmm.12712 (PMC4717849; doi:10.1111/jcmm.12712)

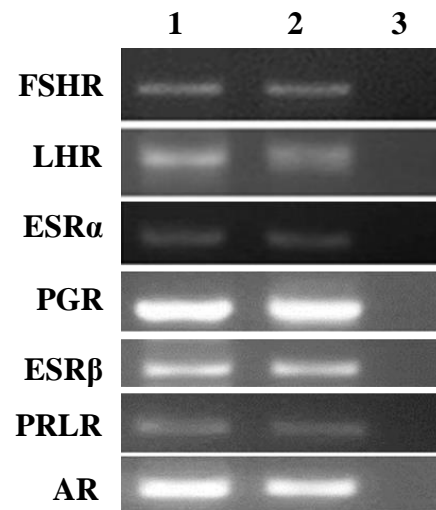

Supplement: Supplementary file 2 — Figure S2 Human CD34+ cells purified from peripheral blood (PB) express functional SexH receptors. RT‐PCR (n = 2) for the expression of pituitary and gonadal SexH receptors after mRNA purification from PB CD34+ cells from a normal donor (Lane 1) and a mobilized donor (Lane 2). Samples containing water only instead of cDNA were used as negative controls (Lane 3). Representative agarose gel of RT‐PCR amplicons is shown. [file JCMM-20-134-s002.pdf]

**A**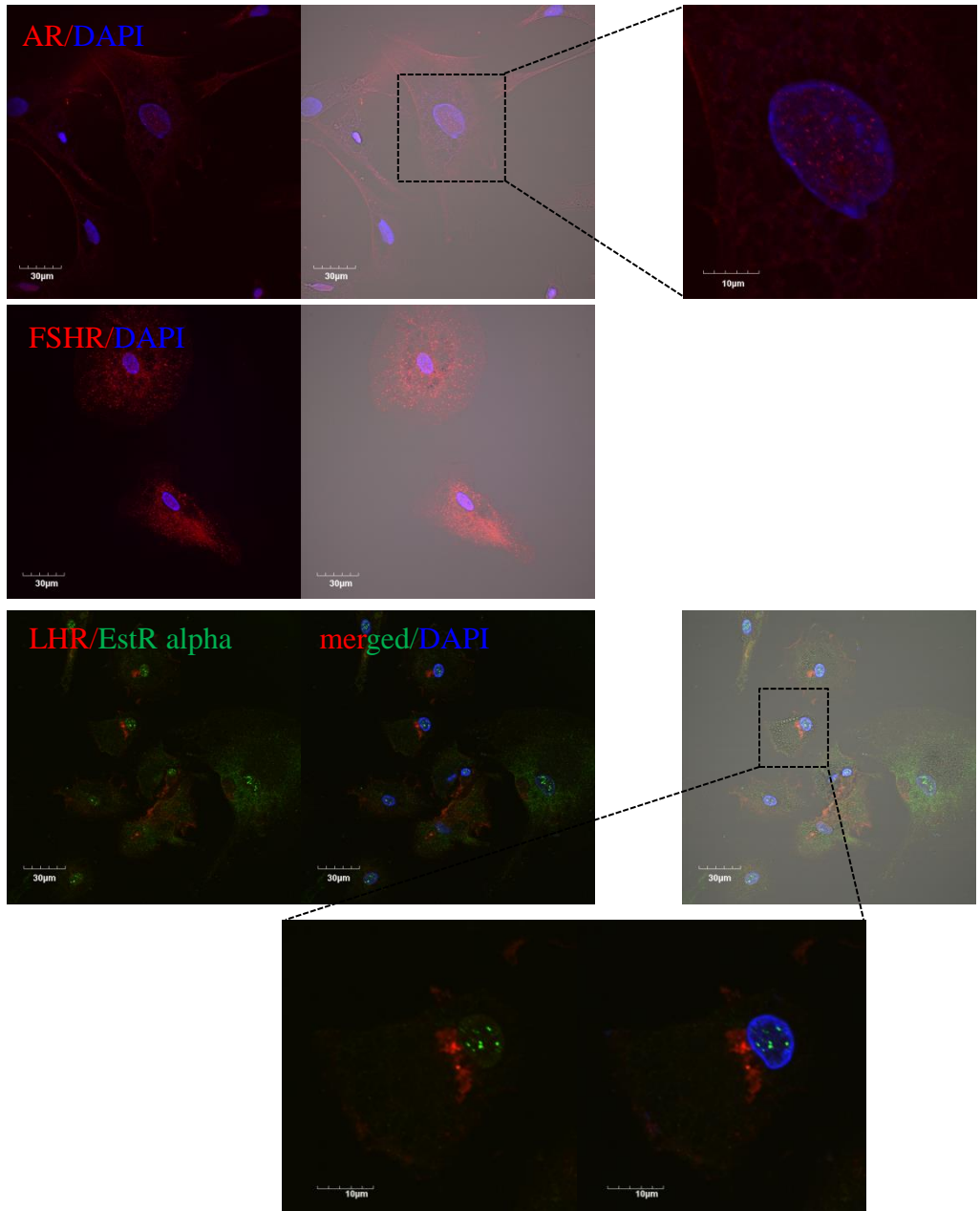**B**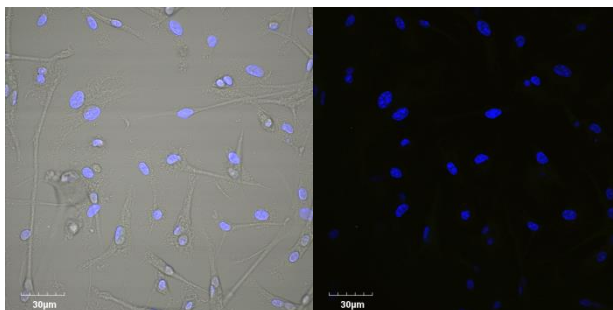

Supplement: Supplementary file 3 — Figure S3 Expression of two pituitary SexH receptors, FSHR and LHR, and two gonadal SexH receptors, AR and ESR, was detected on human MSCs by immunofluorescence staining (A) (n = 2). An example of control staining showing that a secondary antibody conjugated with Alexa 488 or Alexa 594 did not bind to cells if the primary antibody was not employed (B). [file JCMM-20-134-s003.pdf]
